# The fitness costs and benefits of hunter-gatherer locomotor engagement

**DOI:** 10.1017/ehs.2025.10025

**Published:** 2025-10-27

**Authors:** George Brill, Mark Dyble

**Affiliations:** Department of Archaeology, University of Cambridge, Cambridge, UK

**Keywords:** hunter-gatherer, forager, human evolution, locomotion, human locomotion

## Abstract

Bipedalism is a distinguishing feature of our species and, as such, there has been much interest in the energetic costs and foraging returns of walking and running, especially among hunter-gatherer societies. However, humans routinely exhibit extensive locomotor versatility, with hunter-gatherers consistently also swimming, diving, and climbing. Additionally, the fitness costs and benefits of locomotion extend well beyond energy income and expenditure. Here, we review evidence from over 900 ethnographic documents across a worldwide sample of more than 50 hunter-gatherer societies to examine the fitness costs and benefits of walking, running, climbing, swimming, and diving. We show that the fitness costs and benefits of locomotor engagement consistently extend well beyond energetics to include, for example, currencies of status, protection from hazards, and risks of injury or death. These fitness factors differ in significance between locomotor modalities, with implications for the comparison of bipedal and non-bipedal locomotion. For example, while energetic demands represent the major cost of most bipedal engagements, the fitness implications of potential fall injuries may outweigh those of energetics in tree climbing. These results inform existing debates relating to hominin locomotor evolution and hunter-gatherer behavioural ecology.

## Graphical abstract

**Figure:**
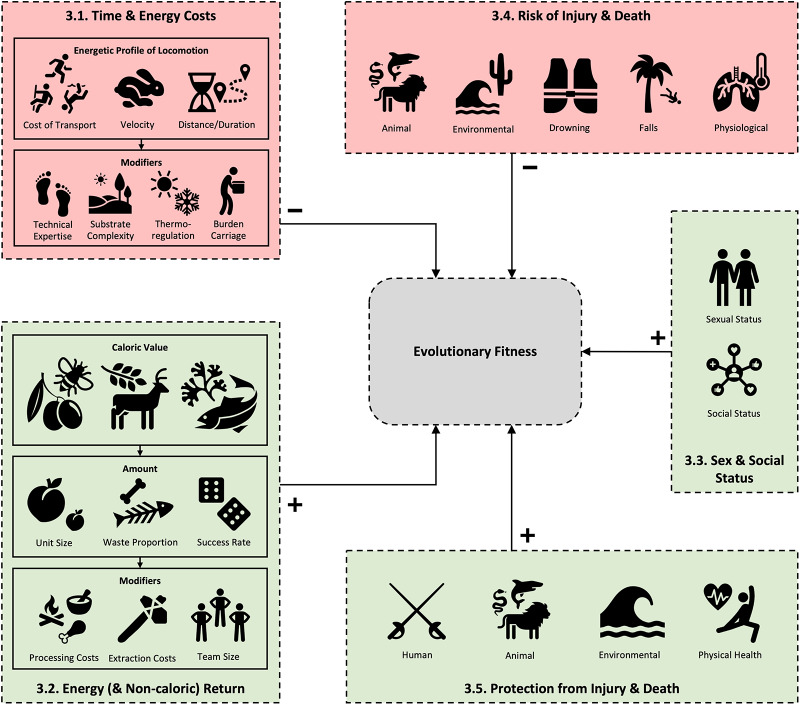

## Social media summary

Exploring the fitness costs and benefits of hunter-gatherer walking, running, climbing, diving, and swimming

## 1. Introduction

Animals vary greatly in their means of locomotion, and biologists have long been interested in understanding the evolution of this diversity (Edwards, 1977; Irschick & Higham, 2016). With bipedal locomotion being a derived and conspicuous human characteristic, the same has been true in evolutionary anthropology, with much interest in the energetics and biomechanics of bipedal movement (e.g., Foley & Elton, 1998; Kuhn et al., 2016; Pontzer, 2012), particularly in hunter-gatherers (Holowka et al., 2022; Morin & Winterhalder, 2024), with wide-ranging implications from human evolution to contemporary population health (Gurven & Lieberman, 2020; Pontzer et al., 2018).

Humans, however, are not limited to bipedal locomotion, with many societies worldwide exhibiting proficiency in swimming, diving, and climbing (Abrahamsson & Schagatay, 2014; Kraft et al., 2014; Schagatay et al., 2011). In a recent paper (Brill et al., 2024), we conducted a cross-cultural analysis of hunter-gatherer locomotor engagement. Our results demonstrated considerable locomotor versatility across a worldwide sample of contemporary and recent hunter-gatherer societies, with high levels of proficiency in walking, running, climbing, swimming, and diving consistently documented across a broad range of ecologies. This locomotor versatility is present not only in the context of food acquisition, but also across a range of other functional domains including leisure, ritual, travel, and protection.

What are the fitness implications of such wide-ranging locomotor engagement? If we consider evolutionary fitness to be the function of survivorship and reproductive success, including that of kin, there are a variety of ways in which hunter-gatherer engagement in locomotion may act to enhance or reduce each (i.e. produce fitness benefits or costs). Previous work has focused on the energetic costs and returns of walking and running (e.g., Glaub & Hall, 2017; Morin & Winterhalder, 2024; Pontzer et al., 2012; Steudel-Numbers & Wall-Scheffler, 2009), the energetics, returns, and risks of climbing (Elton et al., 1998; Kraft et al., 2014; Pontzer & Wrangham, 2004), and the status benefits of the acquisition of unreliable resources (e.g., Gurven & von Rueden, 2006; Wiessner, 1996). However, additional fitness drivers such as the protective function of locomotion have only rarely been addressed (e.g., Kempf, 2009; Watanabe, 1971), while the fitness costs and benefits of aquatic locomotion in humans have received attention only in the form of hunter-gatherer case studies (e.g., Abrahamsson & Schagatay, 2014) or theoretical arguments (Foley & Lahr, 2014). Additionally, with the exception of Kraft et al.’s (2014) treatment of human tree-climbing, cross-cultural treatises are either non-specific (e.g., Devine, 1985; Watanabe, 1971), or pertain to very specific activities (e.g., Morin & Winterhalder, 2024). In short, the fitness costs and benefits of locomotor engagement among hunter-gatherers have not been comprehensively examined, despite being of importance to understanding the development, interaction, and persistence of locomotor modalities throughout human evolutionary history.

Here, we build on essays by Watanabe (1971) and Devine (1985) as well as a more recent detailed treatment of human arboreal ecology by Kraft et al. (2014) to systematically compile ethnographic evidence that demonstrates the fitness costs and benefits of locomotor behaviour across the full breadth of hunter-gatherer locomotion. Within this, we focus on addressing two main questions. First, what are the fitness costs and benefits of hunter-gatherer locomotor engagement? Second, how do these fitness costs and benefits differ between locomotor modalities?

## 2. Methods

We searched over 900 ethnographic texts to produce a comprehensive ethnographic review of the fitness costs and benefits of locomotor engagement across hunter-gatherer societies. Our sample of 57 hunter-gatherer societies are those included in the Standard Cross-Cultural Sample (SCCS; Murdock & White, 2006) – a global sample of 186 human societies chosen to maximise statistical independence – that met the online Human Relations Area Files (eHRAF, 2022) definitions of ‘hunter-gatherer’ (*n* = 41) or ‘primarily hunter-gatherer’ (*n* = 16) based on at least a cumulative 86% and 56% dependence on foraging, respectively (SCCS variables 203–205: hunting, gathering, and fishing). Although not a complete sample of ethnographically documented hunter-gatherer societies, the SCCS was chosen for its global representativeness and relative societal independence (Gray, 1996; Murdock & White, 2006).

We conducted a keyword search of the ethnographic literature obtained from the eHRAF database (eHRAF, 2022; as of August–November 2022). Additional relevant literature was found through GoogleScholar; evidence from a few further societies was included where notably relevant (e.g., Tarahumara ethnographies provide detailed insight into long-distance running; Lieberman et al., 2020). All relevant quotes found can be viewed in data sets S1–7, with further details of keywords and search methodology provided in Brill et al. (2024).

**Figure 1.**
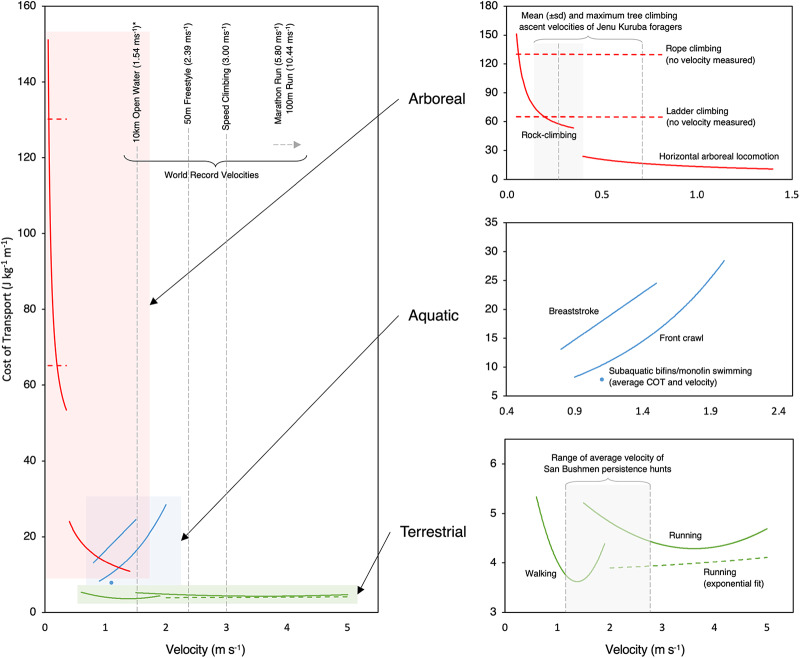
Comparative plots of the mass-specific cost of transport (COT) of various modes of human locomotion against velocity. See Table S1 for data references and calculations. World records (male) as of January 2023 (FINA, 2023; iFSC, 2023; World Athletics, 2023). *Note that the 10 km open water swim represents an approximate average of winning times because records are not recorded. Jenu Kuruba tree climbing velocities from Kraft et al. (2014); San Bushmen persistence hunt velocities from Liebenberg (2006). It should be noted that most values used here represent optimal ‘laboratory’ conditions with trained athletes, and thus the extrapolation to in-situ contexts (as for forager locomotor engagements) should bear this idealism in mind (Devine, 1985; Irschick & Garland, 2001). Indeed, if, for example, we compare actual published data of Hadza men walking at 158 J min^−1^ kg^−1^ (Kraft et al., 2021) at a mean pace of 3.6 and 4.4 km h^−1^ (Marlowe, 2010, p. 121; Pontzer et al., 2015), we calculate values of COT at 2.2–2.6 J kg^−1^ m^−1^ – very different to the ∼4 J kg^−1^ m^−1^ presented in Figure 1.

To provide base energetic expenditure values by which to situate the discussion on energetic costs and calculate caloric return on investment profiles for locomotor subsistence activities, cost of transport (COT; energetic cost per metre of locomotion) values for each locomotor modality were sourced from published respirometry data (see Table S1 for references and standardizations). Figure 1 displays comparative COT traces in relation to velocity for each locomotor modality.

## 3. Results

**Figure 2.**
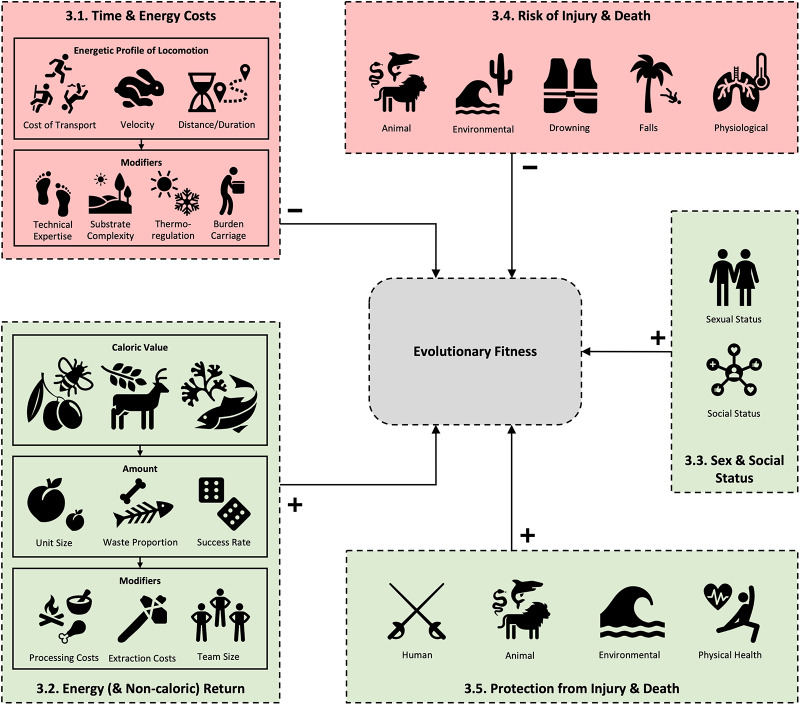
Overview diagram of the categories of fitness costs and benefits, and their subcategories, of hunter-gatherer locomotor engagement. Locomotor costs in red and benefits in green. Numbers refer to Results sections.

Ethnographic evidence for fitness costs and benefits of hunter-gatherer locomotor behaviour was found across a wide variety of contexts. Figure 2 provides an overview of the general themes identified in the ethnographic literature; the presentation of results that follow is structured accordingly.

### 3.1. Time and energy costs

Locomotor engagement expends finite time and energy that cannot be spent on other fitness enhancing activities, as well as reducing the net energetic return of each subsistence acquisition for which locomotor activity is required. With each locomotor modality possessing a characteristic COT, it is well-established that bipedal locomotion represents the most efficient mode of human locomotion (Elton et al., 1998; Di Prampero & Osgnach, 1986); aquatic and especially arboreal locomotion entail energetic demands roughly 2–5 and 2–25 times higher, respectively (see Fig. 1). However, such standardized ‘laboratory’ COT values are incomplete, with a wide range of additional modifiers influencing the actual energetic costs of locomotor engagement evident in the ethnographic record, as follows.

#### Distance and time

COT must be contextualized in terms of distance (or time) travelled. Table 1 summarizes ethnographic examples of time–distance investment for each locomotor modality. Protracted bouts of terrestrial locomotion are the norm, with even routine terrestrial engagements involving multiple hours and kilometres walked or run, and more extreme examples stretching to hundreds of miles over multiple days. Quantitatively, terrestrial locomotion energetics may greatly exceed a typical daily energy budget: compare, for example, the estimated ∼42 MJ per day for Tarahumara kick-ball racing (Balke & Snow, 1965, p. 297) or the 25.5 MJ per day of the average persistence hunt (51 MJ per person over ∼2 days; see B2, Table S2) to the average 8–14 MJ of total daily energy expenditure of Hadza men (Pontzer et al., 2012). Such vast energetic debts (as well as the inevitable physiological damage caused and associated recovery costs) not only encroach on other necessary energetic investments, but also limit the frequency with which such engagements can be repeated.

**Table 1.** Selection of ethnographic examples of investment in hunter-gatherer locomotor engagements. See data set S1 for expanded list, references, full ethnographic passages and interpretative notes. Quote references refer to enumeration within the data set

Context		Investment		Quote Reference
Run/walk	Society	(km)	Notes	
Group movement	!Kung	16	Day’s travel	1
	Kaska	24−32	Day’s travel	23
	Lengua	16−32	Day’s travel	24
	Omaha	16−24	Day’s travel	25
	Siriono	13−16 (up to 40)	Day’s travel	26−27
	Yukaghir	11−56	Day’s travel	28
Hunting	!Kung	21−35	Persistence hunt (single day). Multi-day hunts also documented	29−31
	Copper Inuit	48	Hunt (single day)	33
	Mbuti	5	Average daily movement during hunting	34
	Siriono	32 (up to 64)	Day’s travel.	37−38
Warfare	Chiricahua	64−120	Day’s travel. 3000 miles in 2 months (av. 50 mi/day) documented	58−59
Maximums and anecdotes	!Kung	64−100 (up to 120)	Day’s travel. Longer distances are multiple anecdotal accounts	1−6
	Aranda	32	Day’s travel (anecdotal account, including pregnancy and birth)	10
	Ingalik	64	Day’s travel	15
	Kaska	64 (129)	Day’s travel. 80 miles (129 km) as anecdotal account	16−17
	Montagnais	97−113	Day’s travel	19
	Paiute	97	Day’s travel (anecdotal account)	21
	Tehuelche	64−80	Day’s travel (anecdotal account)	22
Ritual and leisure	Chiricahua	6	Single run. Two-day all-day runs also documented	41−44
	Paiute	13−16	Single run. Twice daily in female initiation	48−49
	Pomo	6−13	Single run	50−51
	Shavante	Not stated	Multiple runs per day for 22 days	52
	Timbira	1−13	Single run. Log up to 100 kg carried in relay form	53−55
	Twana	<1	Single run	56
	Yokuts	3	Single run	57
Generic	Aleut	37−53	Day’s travel	7
	Andamanese	24	Day’s travel. Up to 2−3 days consecutive	8
	Aranda	48	Day’s travel	9
	Callinago	56	Day’s travel. Two days consecutive	11
	Copper Inuit	48	Day’s travel	12
	Hadza	5−12	Average daily movement	13−14
	Mbuti	7	Average daily movement	18
**Climb**		**(units as stated)**		
General/unspecified	Hadza	10 meters	Mean daily climbing in honey extraction	60
	Hadza	15 m	Single climb	
	Mbuti	2.7% by time	Mean daily time climbing during honey expeditions	61
	Mbuti	Multiple climbs of up to 52 m	Single honey extraction	62
	Mbuti	Up to 30 m	Single climb	63
	Semang	50 m+	Single climb	64
	Maori	12−15 m+	Single climb	65
	Aweikoma	9−15 m	Single climb	66
**Swim**		**(km)**		
Hunting	Pomo	2	Single swim performed to and from island rocks	82
Maximums and anecdotes	Maori	2	Single swim, presumably performed both to and from Mokoia	76
	Tupinamba	7−18	Single swim	77
General/unspecified	Abipon	1	Single swim	72
**Dive**		**(units as stated)**		
Hunting	Bajau	3−25 m deep; 2−9 h of which 40−50% surface intervals	Day’s diving. Performed daily	79−81

Comparatively, hunter-gatherer arboreal locomotion, although representing a higher COT than bipedality, typically involves lesser temporal investment than terrestrial locomotion (see Table 1); usually a matter of less than 100 m ascent/descent, or a few minutes climbing at most. Most aquatic engagements are equally brief in comparison; however, examples of longer activities are documented, for example, 2–9 hours of spearfishing in a day by the Bajau (Schagatay et al., 2011), amounting to an estimated ∼5 MJ average (see D, Table S2) of energetic expenditure. Investment in aquatic locomotion by hunter-gatherer children in play may be large: ‘hours and hours’ among the Marshallese (Erdland & Neuse, 1914, p. 95); and in some cases more than equivalent to the time spent on land, for example, among the Manus (Mead, 1930, p. 28), Bajau (Teo, 1989), and Yokuts in summer (Heizer et al., 1952, p. 155).

#### Velocity

The COT of locomotion is always velocity dependent. For terrestrial gaits, this results in different optimal speeds for running and walking, with regular shifting between gaits often being an advantageous strategy (Mateos et al., 2022; Rathkey & Wall-Scheffler, 2017; Steudel-Numbers & Wall-Scheffler, 2009). Ethnographic accounts suggest the exploitation of such strategies. For example, !Kung persistence hunt velocities (Liebenberg, 2006) span a breadth of walking and running speeds (see Fig. 1) and many ethnographic examples document a walk-run gait or gait alternation during long-distance engagements (e.g., Shavante: Maybury-Lewis, 1967, p. 39; Tarahumara: Lieberman et al., 2020).

Due to high postural costs (i.e. the energetic cost of holding a static climbing position), the most energetically efficient way to climb is to do so as fast as possible (Kozma & Pontzer, 2021), subject to the maintenance of efficient technical competency. Conversely, the COT of aquatic locomotion increases dramatically with increasing velocity (Di Prampero & Osgnach, 1986; Schmidt-Nielsen, 1972; Zamparo et al., 2020) resulting in optimal energetic efficiency at lower velocities. Whereas the postural cost of surface swimming (essentially treading water) may be relatively high, aquatic buoyancy essentially negates postural costs for subaquatic locomotion – a dynamic fine-tuned by some divers with the manipulation of starting lung volume, for example, among the Ama (Hong et al., 1963). This means that low velocity hunter-gatherer diving may be much less energetically demanding than might be assumed. Buoyancy dynamics also allow for periods of intermittent gliding with little to no energetic cost (Biewener & Patek, 2010; Kramer & McLaughlin, 2001), exploited by hunter-gatherers such as the *funado* divers of the Ama (Kita, 1965) or Callinago lobster divers (Du Tertre et al., 1667, p. 18), who use weights to facilitate descent. Sliding down tree-trunks in descent [e.g., Batek (Semang in SCCS; G.B. personal observations, 2018–19)] or the use of skis to slide downhill represent similar dynamics in arboreal and terrestrial locomotion, respectively.

#### Technical expertise

The ethnographic record includes many references to the technical expertise of hunter-gatherer locomotion, often in conjunction with descriptions of high levels of performance and apparent (energetic) ease of motion. Terrestrially, the fluidity of hunter-gatherer walking gaits is frequently noted [e.g., !Kung (Marshall-Thomas, 1959, p. 6), Mundurucu (Von Martius, 1867, p. 2), Aweikoma (Henry et al., 1941, p. 6)] as is the technical astuteness of walking and running through complex terrain [e.g., Mbuti (R. Bailey, 1991, p. 58; Putnam, 1948, p. 325), Yurok (Heizer et al., 1952, p. 155), Aranda (Basedow, 1925, pp. 142–144)]. Given that research among both industrialized and non-industrialized populations shows COT is greatly influenced by technical expertise (Black et al., 2018; Holowka et al., 2022; Wallace et al., 2022), it is likely that the energetic savings of hunter-gatherer terrestrial competence are also significant.

In climbing, it is documented among the Yahgan that it ‘takes long practice and trained dexterity’ to acquire ‘adequate proficiency’ in climbing cliff-faces after cormorants (Gusinde & Schütze, 1937, p. 771); so too a delayed proficiency peak is apparent in Jenu Kuruba tree-climbing in the context of honey hunting (Demps et al., 2012), indicative of a technical learning curve. The wide variation of climbing techniques [e.g., Batek (Endicott & Endicott, 2008, p. 88), Mbuti (Ichikawa, 1981, p. 59), Andamanese (Man, 1932, p. 21), see also Kraft et al. (2014); Watanabe (1971)] and swimming strokes [e.g., Warrua (Turrado Moreno & Muirden, 1945, pp. 63, 178)] detailed in hunter-gatherer ethnographies also stress this significance, with the development of optimal gaits to suit the wide range of locomotor engagement contexts and substrates. Indeed, previous research has shown that technique is especially pertinent in non-terrestrial locomotion, generating vast disparities in COT within climbing (Elton et al., 1998), swimming, and diving (Di Prampero & Osgnach, 2018; Pyne & Sharp, 2014; Samimy et al., 2005).

#### Substrate complexity

Energetically challenging substrates may represent the norm for many hunter-gatherer locomotor engagements: examples range from soft sand, standing water and deep snow to steep, rocky trails, overgrown jungle and even wind so strong ‘that it almost halted a man in his tracks’ [Aleut (Innokentii, 1840, pp. 22–24)]; see data set S2 for a full list of ethnographic examples. In terrestrial locomotion, complex environmental substrates (Damavandi et al., 2017; Grant et al., 2022), obstacles (Holowka et al., 2022; Tuck-Po, 2008), path tortuosity (McNarry et al., 2017; Wilson et al., 2013, 2021), and both positive and negative gradients (Minetti et al., 2002; Scarf, 2007) increase COT – in some cases manyfold. Conversely, snow may sometimes decrease the cost of transport, either by obscuring complex terrain (e.g., Montagnais; McGee, 1961, p. 115), or in enabling sled and ski use – used by many societies (Mason, 1896) and affording vast energetic savings (Formenti & Minetti, 2007). Thus, while some accounts reference snow as halving daily travel distances (e.g., Kaska; Honigmann & Bennett, 1949, p. 99), others detail how it extends both their range and possibility (e.g., Copper Inuit; Usher, 1965, p. 155); consider also the use of frozen rivers as throughways.

Hunter-gatherers are documented to climb a wide variety of substrates. For tree climbing, variation includes differences in tree pitch, diameter, and branching structure [e.g., Batek (Endicott & Endicott, 2008, p. 88), Mbuti (Ichikawa, 1981, p. 59), Andamanese (Man, 1932, p. 21), see also Kraft et al. (2014); Watanabe (1971)], sometimes with multi-staged ascents involving horizontal tree transfers and vine bridges [e.g., Batek (Endicott & Endicott, 2008, p. 90), Mbuti (R. Bailey, 1991, p. 46)]. Rock climbing represents another set of substrate variation (e.g., Yahgan; Gusinde & Schütze, 1937, p. 771). Considering the significance of substrate type and route complexity on COT identified elsewhere (Baláš et al., 2014; Booth et al., 1999; Halsey et al., 2016; see Fig. 2), such differences should be assumed for hunter-gatherer climbing. Aquatically, rough waters and currents – frequently documented in hunter-gatherer engagements (see data set S2 for a full list of ethnographic examples) – may be assumed to vastly alter COT values.

#### Thermoregulation

The energetic costs of thermoregulation during hunter-gatherer locomotion are also relevant, with frequent ethnographic documentation of both sustained cold [e.g., Montagnais (Henriksen, 1973, p. 107; Tanner, 1944, pp. 594, 633), Copper Inuit (Jenness, 1923, p. 38), Yukaghir (Jochelson, 1975, p. 419)] and heat [e.g., !Kung (Marshall-Thomas, 1959, p. 13; Silberbauer, 1965, p. 109), Abipon (Dobrizhoffer, 1822, pp. 34–35), Warrau (Turrado Moreno & Muirden, 1945, p. 63)] during locomotor engagement. Thermo-energetics are typically far more significant for aquatic locomotion: even tropical waters lie below human thermoneutral temperatures – 35.0–35.5℃ (Craig & Dvorak, 1966). Yahgan women will swim in waters as cold as 6℃, often insulating themselves with ‘oil or grease’ and ‘immediately hasten[ing] to the hut fire’ afterwards (Gusinde & Schütze, 1937, pp. 370–372); even the Bajau in waters as warm as 26℃ (Schagatay et al., 2011) are reported to periodically warm themselves in the sun during prolonged periods of spearfishing.

#### Burden carriage

Finally, the addition of load carriage, reported to range from minor burdens to as heavy as 90 kg (see Table S3 for a full list of ethnographic examples), increases the COT of terrestrial locomotion. Carrying children is documented almost universally (Mason, 1896; see data set S3), as is resource relocation (see data set S3). So too in swimming, where many methods of burden carriage are described, especially in the transit across rivers. While pushing rafts or baskets (e.g., Yokuts; Gayton & Anna, 1948, p. 161) may potentially reduce COT through additional buoyancy, activities such as holding a firebrand ‘above the water in one hand while paddling with the other’ (Siriono; Holmberg, 1950, p. 11) surely decrease locomotor efficiency. Energetic demands for the underwater wrangling of seaturtles [e.g., Andamanese (Man, 1932, p. 239), Bajau (G.B. personal observations, 2020)] or fish on the end of a spear tether may also be considerable: ‘I saw two Fijians fighting for half an hour in a rough sea with a turtle’ (Deane, 1921 p. 180); ‘sometimes it might require four or five men to overcome a really big turtle in its natural environment’ (Tippett & Alan, 1968, p. 127).

### 3.2. Energy, nutritional and non-edible return

Caloric returns (food) are perhaps the most obvious of all fitness benefits, with positive energetic balance critical to maintaining reproductive function, health, and ultimately survival. Locomotor proficiency is instrumental to almost all hunter-gatherer resource acquisition (see Table 2). High-proficiency locomotion, in particular, is often required to acquire the highest-return resources, for example, running after big game, diving after seafood and fish, and climbing for honey and fruit. In addition to absolute caloric value, the procurement of specific nutritional elements such as protein, fats and essential micronutrients is also important for health and consequent survival, as well as resources of non-calorific value: raw materials sought for their enabling or easing of caloric return, or for other survival faculty. In every case, the energetic return of a resource must be contextualised by the time and energy required to acquire it; see Table S2 for comparative net return rates for four exemplary locomotor subsistence strategies. In most examples, locomotion represents the bulk of the total energetic cost; however, additional factors such as success rates, extraction/processing costs, and team size are also of relevance, as detailed below.

**Table 2.** Energetic return items of hunter-gatherer locomotor subsistence strategies. Species/context are indicated for each society; (–) indicates where original passage did not specify details. See data set S4 for expanded list, references, full ethnographic passages and interpretative notes. Numbers in square brackets refer to quote enumeration within the data set

Return type		Ethnographic examples by society		
		Run	Climb	Swim and Dive
**Foodstuff (fauna)**	**Small game**	Semang (*rat [28]*), Aranda (*lizard, snake, marsupials [33,37]*), Pomo (*ground squirrel [85]*), Yokuts (*rabbit [88]*), Chiricahua (*rabbit [102]*), Tupinamba (*rat [120]*)	Tiwi (*opossum [185]*), Aranda (*marsupials, various [187−188]*)	Miskito (*iguana [340]*)
	**Medium game**	!Kung (*antelope, warthog [5−6, 11−13]*), Hadza (*hrax [15]*), Mbuti (*antelope [22]*), Semang (*deer, pig [27]*), Vedda (*deer [30]*), Tiwi (*wallaby [31]*), Aranda (*kangaroo [32,38]*), Montagnais (*deer [63−64]*), Micmac (*deer [69]*), Saulteaux (*deer [77]*), Eyak (*porcupine [80]*), Pomo (*deer [86]*), Yokuts (*deer [90−91]*), Paiute (*deer, antelope [93−96]*), Creek (*deer [100]*), Chiricahua (*deer, antelope [103−105, 107−111]*), Warrau (*peccary [114]*), Mundurucu (*peccary [116]*), Siriono (*monkey, coati [117])*, Shavante (*peccary, deer [122]*), Yahgan (*guanaco [128]*)	Mbuti (*monkey, duiker [145, 148]*), Warrau (*peccary [236]*), Siriono (*monkey, coati [242, 245−246])*, Aweikoma (*monkey [257]*), Yahgan (*guanaco [268, 272]*)	
	**Large game**	!Kung (*antelope [1−3, 7−12, 14]*), Mbuti (*antelope [22]*), Yurok Samoyed (*reindeer [24−26]*), Gilyak (*elk [41]*), Yukaghir (*elk, reindeer [43, 46]*), Ingalik (*moose, caribou [50−51, 53*), Copper Inuit (*caribou [56]*), Montagnais (*moose, caribou [59−60, 62−63, 65]*), Micmac (*elk, moose [66, 68, 70−72]*), Saulteaux (*elk, moose, caribou, buffalo [73−76]*), Slave (*caribou, elk [78−79]*), Pomo (*elk [86]*), Paiute (*buffalo [92]*), Omaha (*buffalo [99]*)	Kaska (*moose [212]*)	
	**Very large game**	!Kung (*giraffe [3]*), Mbuti (*elephant [20−21]*)		
	**Large carnivores**	Copper Inuit (*polar bear [55, 58]*), Creek (*bear [100]*), Tupinamba (*jaguar [119]*)	Yokuts (*bear [227]*), Warrau (*jaguar [237]*)	Miskito (*alligator [339]*)
	**Eggs**		Vedda (*hornbill [180]*), Tiwi (– *[184]*), Aranda (– *[188]*), Aleut (– *[209−210]*), Yahgan (– *[266]*)	
	**Birds**	!Kung (*francolin [12]*), Eyak (*geese [81]*), Pomo (*road runner [84]*), Callinago (*hummingbird [113]*), Warrau (*- [115]*), Yahgan (*duck [127]*)	Semang (*hornbill,* – *[156,1 64]*), Aranda (– *[187]*), Maori (– *[195−199, 201]*), Aleut (– *[208, 210]]*), Pomo (– *[222]*), Yokuts (*pigeon [228]*), Warrau (*parrot [240]*), Yahgan (*cormorant [269]*)	Paiute (– *[337]*), Lengua (– *[352]*)
	**Large birds**	!Kung (*ostrich [12]*), Aranda (*emu [36]*)		
	**Honey**		!Kung *[129, 133]*, Hadza *[134−136, 141, 143]*, Mbuti *[144, 146, 148−150]*, Semang *[155, 157, 161−162, 166]*, Andamanese *[168−171, 173, 175]*, Vedda *[177−179, 181−182]*, Tiwi *[183, 186]*, Aranda *[187−188]*, Miskito *[233]*, Shavante *[251]*, Aweikoma *[253, 256]*, Lengua *[259−260]*, Abipon *[261−263]*	
	**Insects**		Mbuti (*termites, silkworm [146]*), Aranda (*witchedy grub [188−189]*)	
	**Fish (incl. eels)**	Yurok *[82]*		Semang *[274]*, Andamanese *[275]*, Bajau *[279−282, 285−291]*, Manus *[294−296, 299]*, Mbau Fijians *[305*], Maori *[309]*, Marshallese *[315−317, 319]*, Pomo *[323]*, Yokuts *[332−335]*, Paiute *[336]*, Callinago *[343]*, Tupinamba *[346−349]*, Lengua *[350−351, 353]*
	**Turtle/tortoise**	Miskito *[112]*		Semang *[273]*, Andamanese *[277]*, Bajau *[286, 292]*, Mbau Fijians *[300, 303, 306−307]*, Miskito *[338]*, Trumai *[345]*
	**Sea mammals**	Aleut (*seal [54]*)		Marshallese (*porpoise [318]*), Pomo (*seal, sea lion [322, 324−327, 329]*)
	**Shellfish/other seafood**			Andamanese (*clam [276]*), Bajau (*clams, snails, sea cucumber [286−287, 290]*), Manus (– *[297−298]*), Mbau Fijians (*sea cucumber, shellfish [301−302]*), Maori (*shellfish, paua [308, 310−311]*), Pomo (*mussels, clam [327, 330−331]*), Yahgan (*sea eggs, mussels, sea urchins [355, 357]*)
	**Seaweed/algae**			Bajau *[290]*
	**Crustaceans**			Bajau (– *[290]*), Mbau Fijians (*lobster [304]*), Maori (*crayfish [312−314]*), Callinago (*lobster [341−343]*), Tupinamba (*crayfish, shrimp [349]*), Yahgan (*crab [354]*)
	**Scavenged carcasses**		!Kung *[130]*, Hadza *[142]*	
**Foodstuff (flora)**	**Baobab**		!Kung *[131−132]*, Hadza *[137, 139−140]*	
	**Pine nuts/acorns**		Haida *[214]*, Twana *[216]*, Yurok *[218]*, Pomo *[220]*, Yokuts *[223−226]*, Paiute *[229]*, Aweikoma *[254 − 255,258]*	
	**Coconut**		Andamanese *[176]*, Manus *[190]*, Mbau Fijians *[193−194]*, Marshallese *[202−205]*	
	**Fruits/berries**		!Kung *[133]*, Hadza *[138]*, Mbuti *[151]*, Semang *[154−155, 157, 159, 161−162, 165]*, Andamanese *[169, 171−172]*, Tiwi *[186]*, Aranda *[187]*, Maori *[199−200]*, Marshallese *[202−203]*, Bellacoola *[215]*, Mundurucu *[241]*, Siriono *[244, 247−248]*, Aweikoma *[252]*	
	**Seeds/nuts**		Mbuti *[146, 152−153]*, Tiwi *[186]*, Aranda *[187]*, Mundurucu *[241]*	
	**Fungi**		Tehuelche *[264]*, Yahgan *[265, 270]*	
	**Other**		Warrau (*palm shoots [238]*)	Pomo (*tules [328]*)
**Non-edible resources**	**Skins**	!Kung (*cheetah [4]*), Ingalik (*fox, otter [47−48, 52]*), Montagnais (*otter [61]*), Chiricahua (*deer, – [106, 109]*)		
	**Pets**		Pomo (*eagle [219]*), Callinago (*parrot [235]*), Warrau (*songbirds [239]*), Tupinamba (*various [249]*)	
	**Fibres/vines**		Semang (*rattan [158, 161, 163, 167]*), Andamanese (*anadendron [174]*), Mbau Fijians (*coconut [191−192]*)	
	**Sap**		Semang (*arrow poison [160]*), Miskito (*rubber [232]*)	
	**Feathers**	Chiricahua (*eagle [101]*)	Gilyak (*eagle [206]*), Chiricahua (*eagle [231]*), Tupinamba (*various [249]*)	
	**Bark**		Ingalik *[207]*, Saulteaux *[211]*, Kaska *[213]*, Kutenai *[230]*, Yahgan *[267]*	
	**Wood**		Yurok (*sacred firewood [217]*), Pomo (*firewood [221]*), Yahgan (*firewood [271]*)	
	**Shells/pearls**			Bajau (*pearls [278, 286]*), Manus (*ornamental shells [293]*)
	**Flowers**		Callinago (*white orchid [234]*)	

Terrestrial locomotion is central to most foraging globally, with the majority of each society’s caloric yield dependent on walking mobility, even where other locomotor modalities represent the critical finale. Short-range sprints are frequently reported in the capture of all manner of small animals and some larger game, the latter ranging from seals to emu and elephant. Long-distance running and ‘half running’ (Montagnais; Henriksen, 1973, p. 28) is also documented in many societies in the scouting, encounter, and acquisition of highly mobile larger game (Fletcher et al., 1911, pp. 279–280; Osgood, 1958, p. 253), representative not only of the highest caloric return items acquired by most hunter-gatherer societies, but also a critically important source of fatty food. Our data support recent evidence for the widespread prevalence of persistence hunting (Brill et al., 2024; Morin & Winterhalder, 2024), with ethnographic data identifying targets to include not only medium to large ungulate species, but also include small game and carnivores, even as large as polar bear (see Table 2 and data set S4). The energetic return potential of persistence hunting is extremely large, representing orders of magnitude above that inherent to other locomotor subsistence strategies (see Table S2). Long-distance hunting and travel in fur acquisition and trade is also documented to represent a major means of economic income to some societies [e.g., Slave (Honigmann, 1946, p. 100), Yukaghir (Gurvich & Friedrich, 2020)].

Climbing for subsistence resources is well documented in tropical forest biomes, with tree-top resources such as honey and fruit frequently representing extremely favourable cost to return ratios (Table S2; see also Endicott, 1984; Ichikawa, 1981). While climbing often represents only a very small proportion of a foraging expedition by both time and energetic cost (only 7.9% of the total locomotor cost of Mbuti honey collection, for example; see Supplementary Material), all acquisitions therein are typically entirely dependent on its proficiency. In the case of some rainforest societies, arboreally procured resources may even account for a majority of caloric return, at least seasonally: for example, 70–80% from honey among the Mbuti (Ichikawa, 1981) and almost exclusively fruit among the Batek (Semang in SCCS; Endicott, 1979; Tuck-Po, 2005), each for multiple months a year. The caloric significance of arboreal resources outside of tropical forests should not be underestimated, however, with climbing for honey (a resource of extremely high caloric value) occurring more broadly (see Table 2; see also Marlowe et al., 2014), as well as for a large range of other calorie-rich arboreal resources including arthropods, nuts, seeds, and berries (most notably pine nuts, acorns, coconut, and baobab, each representing dietary staples in many societies for months at a time) also targeted (see Table 2). Climbing also enables access to nesting birds and eggs, as well as arboreal game, such as monkeys, and as a vantage point from which to hunt land-based animals such as duiker and guanaco. Finally, climbing is documented to be prerequisite to several economically important non-edible resources, both directly, as in the case of trade products (e.g., rattan and rubber), and indirectly, as in climbing for coconuts as raw materials for cord and net manufacture, or bark collection for boat building (see Table 2).

While many aquatic resources may be gathered via terrestrial locomotion (often with the employment of technological aids such as nets), acquisition means involving aquatic locomotion are common in hunter-gatherer societies worldwide. Swimming is documented to enable a range of fishing practices, as well as the hunting of large sea mammals (e.g., Pomo; Loeb, 1926, pp. 164, 169, 182). More common is subaquatic locomotion, with marine and freshwater diving utilized worldwide in both the gathering of plants and invertebrates and for underwater hunting whereby various methods of subaquatic spearfishing and netting generate significant (in some cases almost exclusive) caloric returns for some societies [e.g., Bajau (Nimmo, 2000; Sather, 1997, p. 118), Manus (Gustafsson, 1992, p. 183; Province & Carrier, 1982, p. 58), Marshallese (Krämer et al., 1938, p. 176); see also Table S2]; among the Palanan Agta, 62.3% of caloric return is dependent on diving (Dyble, 2016). Diving after larger animals is also documented, including turtles, mahi-mahi and porpoises, iguana, and even alligator (see Table 2), with potential returns being orders of magnitude higher than those of spearfishing (see Supplementary Material).

#### Extraction and processing costs

Energetic costs of resource extraction and processing also decrease net returns. For example, honey collection may require chopping open hives with an axe (e.g., Mbuti; Ichikawa, 1981) whereas game animals must be butchered; the energy cost of tuber digging (see Supplementary Material) represents the major component of a Hadza women’s daily energy expenditure (Kraft et al., 2021). Should resource acquisition occur away from camp, the distance (and burden) of relocation adds to the energetic cost of locomotion involved. For example, ‘the successful [Mundurucu] hunter often ended his day by carrying a 100 pound [∼45 kg] wild pig on his back for three hours on the homeward trail to the village’ (Murphy, 1954, p. 18); similar treks with large carcass weights are reported among the !Kung (Lee, 1979, pp. 223–226) and Barama Carib (Gillin, 1936, p. 9); see also Table S3. Sometimes the task was carried out by another individual altogether: women or girls among the Yukaghir (Jochelson, 1975, p. 122) and Micmac (Denys, 1908, p. 404), for example – with round-trips in both cases amounting to as much as 40 miles. To reduce transport costs the Aleut are reported to herd sealions overland – sometimes taking over 3 weeks – to killing grounds nearer the village (Elliott, 1886, pp. 333–338, 363–370); chasing animals towards home is also documented in persistence hunts (Morin & Winterhalder, 2024). Technology may significantly reduce relocation costs, such as in the use of watercraft on diving expeditions, where transport between dive site and village drastically undercuts the cost of swimming there and back as well as enabling a far larger catch to be transported at negligible additional cost.

#### Team size

The number of individuals required to acquire resources further divides returns (see Table S2). For example, !Kung persistence hunts are documented to involve three to four individuals (Liebenberg, 2006), while among the Chiricahua party sizes would sometimes include a ‘large number’ of individuals to enable a relay-style hunting method, although parties of 1–3 were more common (Opler, 1941, p. 319). These data are in line with Morin and Winterhalder’s (2024) ethnographic review of 71 persistence hunts that calculates a mean team size of 1.6. Honey hunting, while sometimes carried out alone, is also often conducted in ‘small teams’ as among Mbuti (Ichikawa, 1981), see also Vedda (Spittel, 1945, pp. 88–89); among the Hadza, honey hunting groups commonly included two to three individuals (Marlowe, 2010, p. 227). Communal herding efforts are typically documented to involve large numbers of people, often including women and even children, both in terrestrial contexts [e.g., Mbuti (Turnbull, 1965, p. 154), Eyak (Birket-Smith & De Laguna, 1938, p. 112), Aleut (Elliott, 1886, pp. 333–338)] and aquatic [e.g., Marshallese (Erdland & Neuse, 1914, pp. 42–43), Maori (Firth, 1959, pp. 224–225), Mbau Fijians (Deane, 1921, pp. 174, 180; Tippett & Alan, 1968, p. 127)). In each case total energetic return must be divided by the number of individuals involved (even before any further distribution to others not involved), traded against the greater success rates and yield that teamwork provides (Morin et al., 2024; Winterhalder, 1981).

### 3.3. Sex and social status

Locomotor engagement may represent a means to gain social status and sexual eligibility in several ways. Most well recognized is its role in high-value and/or high-status resource acquisition, a relationship documented in ethnographic examples including big game hunting in the Hadza and San (Hawkes et al., 2001; Marlowe, 2004; Wiessner, 2002), honey-climbing in the Sekai and Mbuti (Ichikawa, 1981; Kraft et al., 2014), cliff-side cormorant catching in the Yahgan (Gusinde & Schütze, 1937, p. 236) and turtle hunting in the Meriam (Bird et al., 2001). These examples are in line with a large body of literature on resource acquisition mediated status and intersexual mate choice in many hunter-gatherer societies (Gurven & von Rueden, 2006; R. Kelly, 2013; Wiessner, 1996). In many of these cases, success is dependent on locomotor proficiency.

Non-edible resources may also be of status and associated fitness relevance, such as shells dived for and used as currency among the Manus (Carrier & Carrier, 1989, p. 102; Mead, 1930, p. 56). The use of these same shells, as well as feathers – for example, the eagle feathers climbed after by various North American societies; see Table 3 – for decorative purposes also has status implications, as well as potentially the coveted skins for which it is reported some animals are run down, such as cheetah or fox (see Table 3). The acquisition of the ritually significant anadendron plant by the Andamanese (see Table 3), ‘rare commodities’ by the Pomo (Barrett & Samuel, 1952, p. 272) and ‘charms’ such as a white orchid by the Callinago (Taylor, 1938, pp. 150–151) may also indicate the potential for status gain that is dependent on climbing.

**Table 3.** Ethnographic examples of locomotor engagement with significance to sexual and social status. See data set S5 for expanded list, references, full ethnographic passages and interpretative notes. Numbers in square brackets refer to quote enumeration within the data set

Status gain type	Ethnographic examples by society		
	Run	Climb	Swim and Dive
**SEXUAL SIGNIFICANCE**			
**Relevance to marriage choice**	Timbira (*foot racing for wives [14]*), Saulteaux (*military running renown [11]*)	Semang (*marriage ceremony questioning [21]*)	
**SOCIAL SIGNIFICANCE**			
**Warfare**	Saulteaux *[11]*		
**Hunting**	Yukaghir *[18−20]*, Copper Inuit *[5]*, !Kung *[1]*		
**Acquisition of leadership position**	Yukaghir (*lead hunter [19]*)		Callinago (*prerequisite to chieftainship [22]*)
**Ritual activity**	Chiricahua (*catching coyote on foot [4]*), Tupinamba (*ritual pursuit of captives [17]*)		Pomo (*swims in ritual locations [24]*), Twana (*swims in ritual locations [25]*)
**Sport**	Timbira (*foot races [14]*), Klamath (*foot races [8]*), Shavante (*log races [13]*)		
**Non-specific recognition**	Timbira *[15−16]*, Ingalik *[6]*, Saulteaux *[12]*, Montagnais *[9−10]*, Klamath *[7]*, Chiricahua *[2−3]*		Klamath *[23]*

In other cases, social and/or sexual status gain may be dependant directly on locomotor performance itself. Consider, for example, the praising of a young !Kung girl’s ‘so much “run”’ in pursuit of a young kudu (Shostak, 1981, pp. 101–102), or the prerequisite affirmation of climbing ability for Sekai marriage (Table 3). More general examples of the social significance of locomotor prowess are common ethnographically: for example, the Saulteaux are reported to ‘value speed of foot as highly among their people as the Greeks did in their Achilles’ (Kohl & Wraxall, 1860, p. 122), with ‘even the Indian girls dream[ing] at times that they will become mighty runners, and evince[ing] a pride in excelling in this art, like the men’ (Kohl & Wraxall, 1860, pp. 125–126); for the Yukaghir ‘running itself as part of the hunt is celebrated’ (Jochelson, 1975, p. 126); the Montagnais consider it ‘prestigious to travel long distances in a short time’ (Henriksen, 1973, p. 107); and ‘it is the dream of every Tarahumara youth to become a great runner’ (Bennett et al., 1935, p. 335). More explicitly, the Yukaghir chief hunter was chosen for running ability, and Callinago chieftainship for swimming, diving and burden carriage, among other qualities (see Table 3).

Running prowess is indicated to be significant in both social status and marriage choice among the Saulteaux, while many ritual locomotor engagements are fundamentally rooted in a quest for status, whether explicit or not. For example, engagement in dangerous or difficult locomotor feats for the acquisition of ‘spiritual power’ is documented for both the Pomo and Twana (see Table 3), representative of a social recognition of those ‘sufficiently brave’ to swim across geysers or dive into whirlpools, respectively; the Klamath’s practice of bathing in springs frequented by biting ‘crabs, snakes and other reptiles’ is socially recognized ‘to be of great influence on character and personal courage’ (Gatschet, 1890, p. 181).

Running down a coyote was once prerequisite to manhood status among the Chiricahua, while the Tupinamba assign lifelong titles to those successful in the running down of prisoners (see Table 3). Sport represents perhaps the most obvious link between locomotion and status, with footraces and their runners celebrated by many societies (e.g., Nabokov, 1981), and in the case of young Timbira men, a quite literal competition for ‘beautiful young girls for wives’ (Nimuendaju & Lowie, 1946, p. 144). With the majority of societies examined engaging in some form of locomotor sport or leisure activity, it is perhaps notable that some have theorised the origin of sport as an evolved cultural mechanism for status, display, and (inter- and intra-) sexual assessment (Furley, 2019); a positive association between sporting prowess and reproductive indicators is well documented in industrialized populations (Longman et al., 2015; Postma, 2014; Schulte-Hostedde et al., 2008).

### 3.4. Risk of injury and death

Many hazards are associated with hunter-gatherer locomotor engagements (see Table 4): pathophysiologies and inherent dangers of locomotion itself, so too the hazardous terrain and dangerous fauna of the environments they traverse – risks often well recognized by the societies themselves – with the resultant mortality and morbidity quotient of such risks affecting long-term fitness via their implications for future kin provisioning and reproduction. In each case, the magnitude of fitness cost that each risk represents is the product of its hazard (how seriously its occurrence affects mortality and/or morbidity) and its probability (the likelihood of its occurrence). In some specific cases, the risk profile forms the basis for the social status the activity promises, as discussed above.

**Table 4.** Ethnographic examples of risks associated with locomotor engagement. Note that dangers under running include those of terrestrial locomotion generally. See data set S6 for expanded list, references, full ethnographic passages and interpretative notes. Numbers in square brackets refer to quote enumeration within the data set

Risk type	Ethnographic examples by society		
	Run	Climb	Swim and Dive
**Animal**	Aweikoma (*snake [2]*), Barama Carib (*snake, insects [3]*), Copper Inuit (*polar bear [4]*), Lengua (*snake, alligator, jaguar, tiger [6−9]*), Mbuti (*elephant [10−11]*), Mundurucu (*peccary [12]*), Omaha (*buffalo [14]*), Siriono (*insects, jaguar, alligator, snake [15]*), Warrau (*snake, alligator, fish, jaguar [16−17]*), Yurak Samoyed (*bear [19]*)	Andamanese (*snake [25−26]*), Hadza (*leopard, snake [32, 35]*), Lengua (*jaguar, wasps [37]*), Mundurucu (*peccaries [40]*), Vedda (*bees [45,48]*)	Andamanese (*shark, fish, Tridacna clam [50−52]*), Callinago (*shark [54]*), Klamath (*crab, snake, reptiles [61]*), Manus (*snake, stonefish [62]*), Marshallese (*Muraena eel [64]*), Miskito (*alligator, turtle [66−67]*), Pomo (*shark, orca [68−69, 71]*), Siriono (*piranha, stingray [73]*), Tiwi (*crocodile, jellyfish, shark [74−77]*), Warrau (*snake, alligator, piranha*)
**Environmental**	Aranda (*thorny plants [1]*), Aweikoma (*thorny plants, falling logs, landslide [2]*), Lengua (*thorny plants [6−7*), Siriono (*thorny plants [15]*), Warrau (*mud hole [16]*)	Vedda (*overhead rockfall [47]*)	Marshallese (*coral: suicide [63]*), Mbau Fijians (*waves, coral [65]*), Miskito (*coral [67]*), Pomo (*geysers [72]*), Twana (*whirlpool [78]*), Warrau (*waves [80]*)
**Falls**		!Kung *[20−22]*, Ainu *[23]*, Aleut *[24]*, Aranda *[27−28]*, Aweikoma *[29−30]*, Eyak *[31]*, Hadza *[33−35]*, Ingalik (*suicide [36]*), Marshallese (*incl. suicide [38−39]*), Semang *[41−43]*, Siriono *[44]*, Vedda *[46−47]*, Yahgan *[49]*	
**Drowning**			Bajau *[53]*, Gros Ventre *[55−57]*, Haida *[58]*, Ingalik *[59]*, Tiwi *[77]*, Warrau *[79]*
**Physiological**	Yukaghir (*respiratory damage [18]*), !Kung (*dehydration/heat stroke [5]*), Pomo (*overexertion [13]*)		Klamath (*barotrauma? [60]*)

#### Animal and environmental

Locomotor engagement exposes hunter-gatherers to a wide variety of dangerous fauna (see Table 4). Locomotor subsistence strategies directly target all manner of dangerous prey, including various bear species – ‘it frequently happens that such daring costs him his life’ (Yurok Samoyed; Islavin, 1847, p. 50) – jaguar – to which Lengua ‘not unfrequently lose their lives’ (Grubb et al., 1911, p. 87) – tiger, elephant, peccary, alligator, crocodile, and biting turtle; among the Omaha a ritual documents boys running into panicked herds of buffalo ‘dodging in and out among the animals and [mounted] hunters, for they must take the tongue from a buffalo before it had been touched with a knife’ (Fletcher et al., 1911, p. 282). Incidental interactions with these same animals are reported to be equally hazardous, as well as with poisonous insects and venomous snakes. Swimming and wading across swamps and rivers expose hunter-gatherers to piranha and stingray, while swimming and diving in the open sea brings sharks, killer whales, jellyfish, and other dangerous fauna. Exposure to aquatic parasites and waterborne and mosquito-transmitted diseases is also a potential cost of aquatic locomotion (Kempf, 2009). Arboreally, honeybees are a necessary danger of honey climbing – sometimes potentially deadly (J. Bailey, 1861, p. 290; Spittel, 1945, p. 88); anaphylaxis risk is also relevant (Brown & Tankersley, 2011) – while climbing also exposes one to poisonous snakes, dangerous both inherently and as an instigator of falls.

Environmental risks of locomotor engagement are also well documented, ranging from lacerating plants to landslides, mud-holes, falling rocks during climbing, and aquatic phenomena such as waves, currents, whirlpools, sharp corals, and even geysers (see Table 4). Suicide dependent on locomotor proficiency is documented in both the Marshallese, via leaping from a palm tree or from coral injury by diving, and among the Ingalik, via hanging from a tree.

#### Falls

In line with previous literature on hunter-gatherer tree climbing (Kraft et al., 2014), we found many ethnographic accounts of falls (see Table 4); in the Aweikoma ‘1 per cent of the total number of deaths [were] due to falls from beehives’ (Henry et al., 1941, p. 162). The fact that much honey climbing was conducted in the dark only increases the risk. Similar reports are found for cliff-side climbing, as detailed in the Yahgan (Gusinde & Schütze, 1937, p. 235) and alluded to in the Aleut and Eyak where cliff-side bird hunting (Innokentii, 1840, p. 400–401) and mountain goat hunting (Birket-Smith & De Laguna, 1938, p. 100), respectively, were considered ‘the most dangerous type of hunting’.

To contextualize fall risks, onto concrete, the chances of survival approach 0% above 5 storeys (∼19 m; Risser et al., 1996). This survival threshold may increase with other landing surfaces, yet even falls of far lesser height may be fatal, and short- and long-term injury or disability also entail major fitness costs. With rainforest hunter-gatherers routinely ascending to heights of 50 m or more (Kraft et al., 2014), and the majority of hunter-gatherer societies that climb doing so to more than 10 m in height (Brill et al., 2024), it is unsurprising that arboreal fall deaths and injuries are well recognized by hunter-gatherer societies themselves, often documented in taboos, beliefs, and mythologies [e.g., Marshallese (Erdland & Neuse, 1914, p. 261), Eyak (Birket-Smith & De Laguna, 1938, p. 100), Maori (Best, 1924, p. 460)]. Indeed, the Ainu and Batek (Semang in SCCS), respectively, viewed falling from trees to be the action of demons (Batchelor, 1927, p. 327) or a spiritually induced punishment (Endicott, 1979, pp. 7, 59, 81, 174), while, to the Aweikoma, it represented the origin of death (Henry et al., 1941, p. 151).

#### Drowning

Drowning or near drowning is documented in multiple societies (see Table 4), inspiring protective ritual offerings among the Gros Ventre (Cooper et al., 1957, pp. 15, 382, 386). Such risks are presumably most relevant for hunter-gatherer divers, with data on industrialized athletes revealing that 10% of competitive freedives involve hypoxic episodes on surfacing, with 6.1% of depth dives resulting in loss of consciousness (Lindholm, 2007; Lindholm & Lundgren, 2009). Although the shallower dives of hunter-gatherers greatly reduce this risk, that spearfishing is often undertaken solo increases the severity of this hazard dramatically.

#### Physiological

Given proportionally greater pressure gradients at shallower depths, the potential for pulmonary barotrauma is significant even during the typically shallow dives of hunter-gatherers, especially if diving on partial lung volumes as some societies are documented to do. An account among the Klamath of haemorrhaging from the mouth and nose as a result of diving into deep pools to seek spiritual power (Spier, 1930, p. 71), ascribed to the actions of water spirits, may potentially represent symptoms of pulmonary or sinus barotrauma as seen in competitive freediving (Bourolias & Gkotsis, 2011; Patrician et al., 2021). Historically the Ama also report a range of diving-related complaints, with ear, nose, and throat issues due to pressure and seawater exposure most common (Harashima & Iwasaki, 1965). Coldwater immersion also carries risks of hypothermia, cardiac issues and subsequent drowning (Tipton & Bradford, 2014), with divers at risk during prolonged exposures even in warmer waters (Craig & Dvorak, 1966; Molnar, 1946).

Terrestrially, among the Yukaghir, an account of ‘bloody foam appear[ing] at the mouths of the hunters’ ‘during very fast runs on snowshoes’ (Jochelson, 1975, p. 126) appears to indicate respiratory damage, perhaps related to the cold conditions run in, although likely temporary and quickly recoverable. In hot climates dehydration and heat stroke are also risks of running engagements (Hora et al., 2020), potentially with life-threatening consequences, as is insinuated in the documentation of San persistence hunts (Liebenberg, 2006); fainting from overexertion is also documented among the Pomo during sport (Loeb, 1926, p. 218).

### 3.5. Protection from injury and death

Just as locomotor engagement may carry injury and mortality risk, it may also represent means to avoid such risks. Higher proficiency in certain locomotor modalities provides inherent protection from passive hazards: for example, the ability to swim well (or at all) reduces the risk of drowning in both intentional and unintentional immersion – not uncommon given the number of canoe-faring societies – while greater climbing proficiency lowers the risk of falling for any given climb. In fact, many of the dangers detailed in the previous section may be mitigated at least to some extent through greater locomotor proficiency: ‘Walking, to the [Mbuti], means being able to run swiftly and silently, without slipping, tripping or falling. Every day he depends for his food on his ability to “walk”, and more than once his life will be saved by the same ability, when he has to run from a charging buffalo or creep away unnoticed from a sleeping leopard’ (Turnbull, 1962, p. 79).

#### Human, animal, and environmental

Running, climbing, swimming, and diving are all documented in the escape of enemies and enemy fire, whether in warfare, intergroup raiding, or within-group violence, while a range of animal species are also reported to be evaded via locomotor proficiency (see Table 5). Regarding animal threat, running is frequently used to escape enraged prey, as well as less obvious threats such as disturbed wasp nests. Climbing trees is reported in the evasion of buffalo, jaguar, and bear attacks, and as arboreal sanctuary in the hunting of moose, gemsbok, and peccary. Interestingly, despite long-standing evolutionary assertions of the potential of aquatic sanctuary from terrestrial predators (Broadhurst et al., 2011; Cunnane, 1980), and (limited) evidence of such among other primates (Kempf, 2009), no ethnographic examples were found. Concerning environmental hazards, treehouses are documented in avoiding floodwaters and roaming tigers, and the survival value of firewood gathering is significant – a task reported to account for much of the walking engagement (and in some cases climbing; see Table 2) of some societies, such as the Aranda and Yahgan.

**Table 5.** Ethnographic examples of risk mitigation or evasion via locomotor engagement. See data set S7 for expanded list, references, full ethnographic passages and interpretative notes. Numbers in square brackets refer to quote enumeration within the data set

Threat type	Ethnographic examples by society		
	Run	Climb	Swim and Dive
**Human**	Abipon *[1]*, Aranda *[3]*, Callinago *[4]*, Chiricahua *[5−6]*, Gros Ventre *[7]*, Maori *[9]*, Mbau Fijians *[10−11]*, Mbuti *[12, 14]*, Mundurucu *[15]*, Pomo *[16−17]*, Saulteaux *[18]*, Timbira *[20]*	Mbau Fijians *[27]*, Mbuti *[28]*, Saulteaux *[30−31]*, Timbira *[33]*	Abipon *[38]*, Mbau Fijians *[39−43]*, Paiute *[44]*, Saulteaux *[45−46]*, Shavante *[47]*, Tupinamba *[48]*, Twana *[49]*
**Animal**	Ainu (*bear [2]*), Kutenai (*bear [8]*), Mbuti (*buffalo, leopard [13]*), Semang (*wasps [19]*)	Ainu (*bear [21]*), Kaska (*moose, bear [22−23]*), !Kung (*gemsbok [24]*), Kutenai (*bear [25]*), Lengua (*jaguar [26]*), Mundurucu (*peccary [29]*), Semang (*tiger [32]*), Tiwi (*buffalo [34]*), Vedda (*bear [35]*), Warrau (*jaguar [37]*)	
**Other**		Warrau (*flooding [36]*)	

#### Physical health

Many societies are documented to subject their children (and in some cases adults) to rigorous training routines and initiation practices involving running, load carrying, and/or swimming [e.g., Chiricahua (Opler, 1941, pp. 67, 74–75, 444), Eyak (Birket-Smith & De Laguna, 1938, p. 162), Paiute (I. Kelly, 1934, p. 162), Timbira (Nimuendaju & Lowie, 1946, p. 144), Klamath (Spier, 1930, p. 71), Saulteaux (Jenness, 1935, p. 94), Aranda (Strehlow, 1947, pp. 107–108), Shavante (Maybury-Lewis, 1967, pp. 119–123)] for the purposes of developing and maintaining good health and physical capacity. While such practices often occur in a ritual or military context, the link to health is also often emphasized by participants [e.g., Chiricahua (Opler, 1941, p. 67), Twana (Smith, 1940, pp. 188–190), and Gros Ventre (Flannery, 1953, p. 144)], especially in the context of coldwater immersion.

While in the context of highly active hunter-gatherers it may seem a null discussion (and indeed at odds to the potential health costs of locomotor engagements vastly exceeding energy budgets; see earlier section), the contribution of regular locomotor engagement to the maintenance of general health and physical fitness in hunter-gatherers is likely significant (Pontzer et al., 2012); indeed, among hunter-gatherers, locomotion may frequently account for large portions of daily energetic expenditure (see Table 1; see also Pontzer et al., 2012). This is in line with more general research linking regular physical activity to a range of health outcomes, from bone density and physical capacity to mental health and non-communicable diseases (Eaton & Eaton, 2003; Warburton et al., 2006) – in turn generating non-negligible implications for evolutionary fitness via future health and faculty.

## 4. Discussion

### 4.1. The fitness costs and benefits of hunter-gatherer locomotor engagement

Ethnographic evidence for a variety of fitness consequences is present for hunter-gatherer engagement in walking, running, climbing, swimming, and diving. The cross-cultural evidence provided here corroborates previous research demonstrating the considerable energetic costs and benefits of bipedal subsistence strategies (Glaub & Hall, 2017; Morin & Winterhalder, 2024), as well as the significance of status gain in locomotor subsistence strategies, as previously indicated by a variety of research concerning big game hunting (Gurven & von Rueden, 2006; Wiessner, 1996). Our data, however, demonstrate that such fitness considerations also extend to arboreal and aquatic locomotion, as well as to a range of functional contexts outside of the quest for food such as leisure and protection. The fitness consequences of these contexts may be equally as important in driving locomotor engagement in hunter-gatherers. Indeed, despite an almost exclusive focus on the energetics of locomotion in much of the literature, it is apparent that, as has been previously identified in the case of human tree-climbing (Kraft et al., 2014; Pontzer & Wrangham, 2004), the fitness consequences of hunter-gatherer locomotion involve more than energy balance alone.

**Figure 3.**
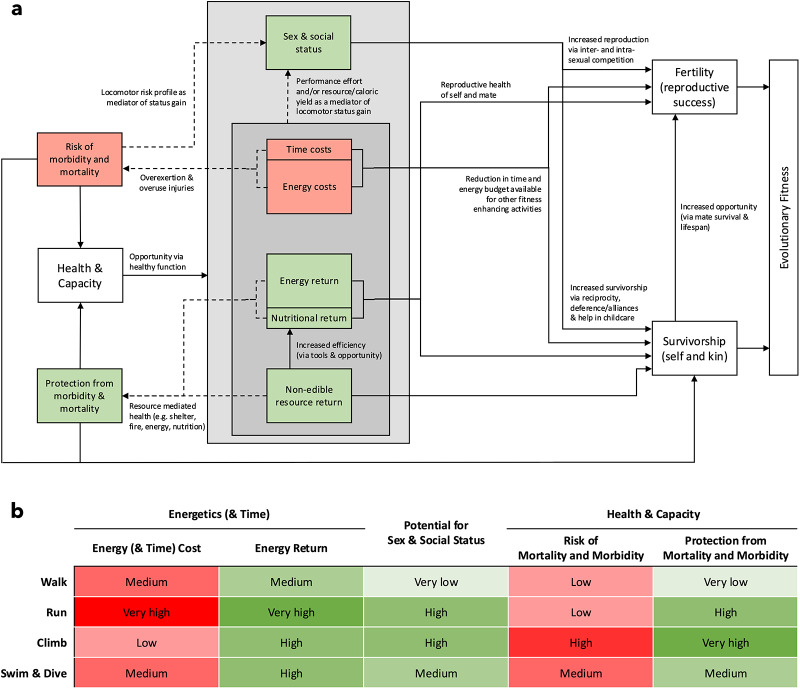
(a), Web of interactions by which locomotor engagements affect evolutionary fitness. Negative fitness effects (costs) are coloured red and positive fitness effects (benefits) are coloured green. Solid arrows indicate direct effects, and dotted lines indicate potential additional associations between elements. (b), Hypothesized comparative significance approximation of typical fitness properties for each hunter-gatherer locomotor modality based on the results presented. Note that energetic elements refer to typical locomotor activity bouts rather than baseline COT. Negative fitness effects (costs) are coloured red and positive fitness effects (benefits) are coloured green; light to dark heatmap represents a 5-point scale from ‘very low’ to ‘very high’.

To ascertain how various fitness costs and benefits influence locomotor behaviour it is necessary to consider the manner in which they interact: profit from resource acquisition or status gain must be balanced against losses due to exposure to hazards, and the time and energy that cannot be spent on other activities that might otherwise increase reproductive fitness (Winterhalder, 1981). Theoretically, any modality that affords a highly favourable fitness cost–benefit ratio will be preferred, increasing engagement and selecting for the locomotor proficiency it involves across both life history and evolutionary timescales. Figure 3A shows this balance, mapping each of the elements discussed to currencies of evolutionary fitness. However, although this is straightforward to map in qualitative terms, to standardize and quantitatively calculate fitness across these elements is challenging, moving from quantifications of caloric return rates alone (e.g., Kraft et al., 2021; Morin & Winterhalder, 2024) to more complex models that integrate for multiple currencies of fitness input (e.g., González-Forero & Gardner, 2018). While such quantification would be extremely difficult to apply to the ethnographic data, even on a qualitative level it can be clearly seen how non-energetic components may alter our energetic-based models of evolutionary fitness. For example, a foraging trip climbing for honey may have the same net energetic return rate to a foraging trip walking several miles to gather tubers but the risks associated with the climbing trip will likely be significantly greater (Kraft et al., 2014; Pontzer & Wrangham, 2004). Conversely, the acquisition of honey may have greater benefits for an individual’s social status than the acquisition of tubers. Solely energetic models miss these important fitness modifiers. This is implicit in previous assertions that falls during climbing may represent a significant enough fitness cost to potentially outweigh energetic efficiency as the primary driver maintaining arboreal competence in *Homo sapiens* (Kraft et al., 2014) – a dynamic likely shared by chimpanzees (Pontzer & Wrangham, 2004).

### 4.2. Variation in fitness costs and benefits between locomotor modalities

When the full set of locomotor contexts and associated fitness consequences are considered, it becomes apparent that the fitness costs and benefits of bipedal locomotion may be very different to those of climbing, swimming, or diving. For example, although higher proficiency in both running and climbing each represent an increased likelihood of hazard mitigation, running faster will provide little extra chance of outrunning faster cursorial predators, whereas climbing more proficiently may well avoid death by falling. Conversely, whereas improved running economy will account for vast energetic savings over a 3-hour persistence hunt, better climbing technique will provide negligible energetic savings across the handful of minutes actually climbing during a honey collecting expedition.

To summarize the comparative findings of the data presented here, terrestrial locomotion represents the largest component of the human subsistence equation. It is the least energetically expensive, at least in terms of cost of transport, and typically incurs little risk beyond that inherent in inhabiting terrestrial environments generally. However, the durations of bipedal engagements are frequently protracted, especially in the case of high-return resources such as large game, amounting to high energetic costs overall, and much time commitment. Running as a subsistence strategy typically represents especially high energy throughput, with high energetic costs balanced by the potential for extremely high returns. Arboreal engagements, while accruing an extremely high cost of transport, provide access to high-value and reliable resources such as honey, fruit, acorns, or pine nuts, often extremely rapidly, meaning that total energetic expenditure is likely much lower than most terrestrial engagements, as well as entailing lower levels of temporal commitment. Climbing also has considerable potential to represent sanctuary from terrestrial hazards. The major cost associated with climbing, however, is the innate hazard, with the risk of mortality and morbidity due to falling likely carrying far more direct and severe fitness consequences than the energetic cost of arboreal activities. Aquatic locomotion also represents a higher cost of transport than bipedal locomotion; however, velocity and distance travelled is typically low, with the exploitation of buoyancy dynamics allowing lower energetic costs than might be expected. As with arboreality, the aquatic and subaquatic environment embodies a reliable source of high-nutrient density resources ripe for exploitation, often quickly and relatively easily, yet also represents a literal physiological death zone while simultaneously exposing hunter-gatherers to a host of aquatic threats. Figure 3B provides a hypothesized comparative significance approximation of the major potential fitness costs and benefits for each locomotor modality based on the ethnographic evidence compiled.

### 4.3. Study limitations

Although a rich and extensive source of information, there are limitations of sourcing data on locomotion (and generally) from the ethnographic record (Brill et al., 2024). Indeed, in addition to potential inaccuracies and exaggerations, each ethnographer’s work is influenced by biases relating to their methodology, personality, engagement context (often colonial), and interests, distorting both their understanding and documentation of their observations (Hayter, 1994; Wobst, 1978). No doubt many relevant anecdotes and phenomena were not observed by or reported to ethnographers, occurred outside of ethnographic coverage periods, or were simply not documented despite observation. As such, a general tendency towards underrepresentation of the elements discussed in this paper is likely, the extent to which must vary from element to element in a manner that is difficult to ascertain. Despite these limitations, however, much of the content discussed in this study is unambiguous (e.g., a fall from a tree), and, short of intentional distortion or misinformation by informants, is difficult to misrepresent. Further, because the study seeks to identify themes and cross-cultural consensus across hunter-gatherer societies and their many ethnographies worldwide, each element discussed is contextualized and cross-referenced, helping to validate specific anecdotes and descriptions, as well as their significance to the degree possible.

## 5. Conclusions

Our results identify the costs and benefits that make up the fitness landscape of hunter-gatherer walking, running, swimming, and diving. The implications for the significance of a broad set of fitness costs and benefits within the human locomotor spectrum are large, with ramifications for both frameworks of hunter-gatherer behaviour, and, with cautious extrapolation, the evolution of diverse locomotor performance in human evolutionary history. First, our data indicate that, even following the evolution of a bipedal morphology, hunter-gatherers routinely utilize non-bipedal locomotor behaviours to exploit extremely profitable arboreal and aquatic niches, as well as to escape threats and gain social capital. The breadth of cross-cultural evidence for each of these elements suggests the continued adaptive significance of non-bipedal locomotion long beyond the shift to ‘obligate’ bipedalism. As such, we argue that the relevance of non-bipedal locomotion to human evolution may have been more significant than is typically assumed. Even for aquatic locomotion, a modality often overlooked in mainstream narratives of human evolution, it is apparent that the discipline embodies many accessible fitness gains, and that contemporary hunter-gatherers are routinely documented to swim and dive, even despite the associated gauntlet of aquatic hazards.

Given the range of ethnographic evidence for both diverse locomotor engagements outside of a subsistence context (Brill et al., 2024), as well as the breadth of fitness properties – both positive and negative – that operate beyond an energetic currency, it is clear that models analysing locomotor fitness cannot be adequately constructed on the basis of energetics alone. So too must it be acknowledged that the relative significance of various fitness costs and benefits may vary dramatically between modalities, and that comparative energetic demands or net energetic return values of different modalities do not tell the whole story, or even necessarily that much of it (Kraft et al., 2014; Pontzer & Wrangham, 2004). In the case of non-bipedal locomotion, it is likely not energetic economy but the mitigation of risk that will be of greatest importance in determining the fitness consequences of engagement.

## Supporting information

Brill and Dyble supplementary material 1 Brill and Dyble supplementary material

Brill and Dyble supplementary material 2 Brill and Dyble supplementary material

## Supplementary material

The supplementary material for this article can be found at https://doi.org/10.1017/ehs.2025.10025.

## Research transparency and reproducibility

The full data set compiled by this study is available in the electronic supplementary material accompanying this article.
