# Supplementary material for: The fitness costs and benefits of hunter-gatherer locomotor engagement
Source: Evol Hum Sci. 2025 Oct 27;7:e36. doi: 10.1017/ehs.2025.10025 (PMC12645331; doi:10.1017/ehs.2025.10025)
Supplement: Brill and Dyble supplementary material 2 — Brill and Dyble supplementary material [file S2513843X2510025Xsup002.docx]

Supplementary Material

# Cost of Transport of Human Locomotion

***Table S1.*** *Experimental data used to construct velocity-specific COT traces for modalities of human locomotion.*

| **Modality** | **Reference** | **Subject details** | **Notes** |
| --- | --- | --- | --- |
| **Terrestrial** |  |  |  |
| Walking | [1] | Male; 72.7kg;  consistent runners | Values established from graph in publication |
| Running |  |  |  |
| Running (exponential fit) | [2] | Male; 70kg |  |
|  |  |  |  |
| **Aquatic** |  |  |  |
| Front crawl | [3] | Male; 75.9kg;  elite college swimmers |  |
| Breaststroke |  |  |  |
| Subaquatic fin swimming | [4] | Male/female; mono/bifins; experienced freedivers; neutral buoyancy; wetsuited | Plot represents mean COT and velocity plotted. No mass given: 65kg used in calculations |
| **Climbing** |  |  |  |
| Horizontal locomotion *(above and below substrate locomotion; leaps and drops)* | [5] | Male; 73.9kg;  elite parkour athletes | Trace calculated from 4^th^ (practiced) trial data |
| Rock-climbing  (*90º wall; no overhangs/ledges; difficulty grade YDS 5.6-5.10)* | [6] | Male/female; 80.1kg;  experienced rock climbers |  |
| Ladder climbing | [7] | Male; 70.3kg;  parkour practitioners | No velocity measured |
| Rope climbing |  |  |  |

# Net Energetic Return Calculations

***Table S2.*** *Comparative energetic costs, returns and net returns for common hunter-gatherer subsistence strategies. See Supplementary Material for full calculations and references. * = presented as mean (range) of 71 ethnographic records of persistence hunts for various prey types [8].*

| **SUMMARY** |  | **Subsistence strategy** | **Reference**  **Society** | **Duration**  **(hr:min)** | **Team size** | **Net Return (MJ)** | | **Return: Cost Ratio** | **Net Return Rate (MJ/hr)** | |
| --- | --- | --- | --- | --- | --- | --- | --- | --- | --- | --- |
|  |  |  |  |  |  | **Total** | **Per person** |  | **Total** | **Per person** |
|  | A | Tuber foraging trip | Hadza | 2:45 | 3.8 adults | - | 14 | 10 | - | 5 |
|  | B1 | Persistence hunting | !Kung | 3:35 | 3 | 1382-2320 | 461-773 | 26-70 | 385-647 | 128-216 |
|  | B2 | Persistence hunting | *Varied | 19:45 (~2d)  (0:18-180:00) | 1.6  (1.0-11.3) | 1123  (-632-18,630) | 877  (-56-18,630) | 33  (-1-542) | 86  (-2-1397) | 74  (>-1-1397) |
|  | C | Honey collecting | Mbuti | 7:00 | 6 (likely split) | 170 | 28 | 16 | 24 | 4 |
|  | D | Spearfishing | Bajau | ~5:00 | 4 | - | 2-59 | 1-13 | - | <1-12 |

| **ENERGETIC COSTS** |  | **Cost of locomotion (MJ)** | | **Cost of extraction (MJ)** | **Cost of relocation (MJ)** | **Cost of processing (MJ)** | **TOTAL (MJ)** | | |
| --- | --- | --- | --- | --- | --- | --- | --- | --- | --- |
|  |  | **Total** | **Per person** |  |  |  | **Total** | **Per person** | **of which**  **locomotion (%)** |
|  | A | - | 0.3 [walk] | 1.2/person | Negligible | Negligible | - | 1.5 | 18 |
|  | B1 | 18 [run/walk] | 6 | - | 9 (combined estimate) | | 27 | 9 | 67 |
|  | B2 | 85 [run/walk]  (1-928) | 51  (1-309) | Proportionally negligible | | | 85  (1-928) | 51  (1-309) | Vast majority |
|  | C | 9 [walk]  0.7 [climb] | 1.5 | 2 total | Negligible | Negligible | 11 | 2 | 82 |
|  | D | - | 5 | - | Negligible | Negligible | - | 5 | 100 |

| **ENERGETIC RETURNS** |  | **Item/s** | **Unit weight (kg)** | **Waste**  **(%)** | **Edible value (MJ/kg)** | **Return profile** | | |  | **TOTAL (MJ)** | |
| --- | --- | --- | --- | --- | --- | --- | --- | --- | --- | --- | --- |
|  |  |  |  |  |  | **Units** | **Success rate** | **Total weight (kg)** |  | **Total** | **Per person** |
|  | A | Tuber | 4.6 | - | 3.3  (inc. waste) | 10.3 | - | 4.6 |  | - | 15 |
|  | B1 | Kudu | 199-314 | 43-46 | 13.1 | 1 | 0.5-0.8 | 199-314 |  | 1409-2347 | 235-626 |
|  | B2 | Varied | 170±207  (2-600) | 26±6  (12-32) | 6.3±1.6  (4-12) | 1.5±3.4  (1-30) | 0.5-0.8 | 249±439  (2.3-3075) |  | 1208±2478  (16-18,665) | 927±2347  (11-18,665) |
|  | C | Honey hive | 4.7 | 25 | 12.7 | 4.1 | - | 18.9 |  | 181 | 30 |
|  | D | Fish | - | 30 | 9.1 | - | - | 1-10 |  | - | 6-64 |

## A. Foraging for tubers (Hadza) [9]

**Return.** Data on over 40 digging expeditions reports a mean weight of 4.6kg (10.3 units) of tubers dug per woman when all species of root are combined [9]. An average (weighted to account for species frequency across all expeditions) of the caloric values by Vincent [9] gives a mean energetic return of 3297kJ/kg: 15,166kJ for the 4.6kg total.

**Cost.** Hadza women conduct root digging expeditions with a mean group size of 3.8 adults plus children of ages 2-12, for a mean total party size of 5.31 [10 p.122]. Suckling babies will be carried on backs while children able to walk will be left in camp or accompany the team on their own feet [9]. The distance to the digging patch is typically less than 5km [9], or 10-30 minutes [10 p.109]. Additional travel between patches may amount to 5-10 minutes of walking for each relocation up to as many as 6-7 patches in total [10 p.109], although follow data collected by Vincent [9] suggests that plants are typically close together with an average of only 3.6 minutes of relocation across the entire expedition. Assuming a 40 minutes round trip to and from camp with an additional 3.6 minutes search time, an average body mass of 46.33kg [10] and COT as 0.137kJ/min/kg (for Hadza women walking out of camp; [11]), the total cost of walking for a Hadza woman is 277kJ. An additional relocation cost of the average 4.6kg of tubers harvested per woman [9] on the 20 minutes trip back to camp represents a negligible additional 12kJ. This assumes no child is being carried and that all tubers are carried back to camp rather than eaten during the expedition. This latter assumption is false, with each woman typically eating a few of her tubers out in the field [10 p.110].

While the cost of tuber processing are negligible—involving ~5 minutes of roasting on a fire followed by hand peeling and chewing [9,10 p.110]—the cost of extraction is significant: ‘Strength and stamina are essential for the 10–20 minutes of vigorous digging needed to expose a big tuber and pull it up’ [10 p.109]. According to data on 40 trips the average digging time was 91.9 minutes across all tuber types [9], with a cost of 290J/min/kg (for Hadza women: [11]). This gives a total extraction cost of 1235kJ for our 46.33kg Hadza woman.

All in this mean foraging expedition amounts to a total of 165.5 minutes total, including the routine half hour of eating and resting reported by Marlowe [10 p.113], and amounts to a total energetic cost of 1512kJ.

**Net.** Overall, this amounts to a net return of 13,654kJ (return:cost ratio of 10:1) over an average of 165.5 minutes for a net return rate of 4950kJ/hr (per person) for Hadza tuber foraging.

## B1. Persistence hunt for kudu (!Kung) [12]

Net return values of !Kung persistence hunts were calculated by Glaub & Hall [12] based on existing data documented for persistence hunts [13] and !Kung hunters [14]. They used average parameters of 215 minutes of running for three 50kg hunters in pursuit of kudu of various sizes with a success rate of 50-80%, adjusting for edible caloric value and an arbitrary relocation/processing cost (results presented in Table S2). Note locomotor cost estimations do not account for ‘the difficult conditions that need to be overcome, including extreme heat, soft sand, and sometimes thick bush’ [13 p.1018].

## B2. Persistence hunting across various species (71 ethnographic accounts worldwide) [8]

## *(Morin & Winterhalder 2024)*

Morin & Winterhalder [8] conducted an ethnographic review of 391 persistence hunts globally, calculating return rates for 71 instances ranging from small game to moose. They modelled hunting costs based on laboratory values for human walking and running, adjusted for a model hunter of 50kg and increased by a factor of 1.3 to account for more difficult terrain in the field. A 50-75% success rate was used. Processing costs were not incorporated into the model, but may be considered negligible compared to the magnitude of locomotor costs and returns present in most persistence hunts. We used Morin & Winterhalder’s [8] raw data to calculate energetic costs and returns, both total and per individual, as well as net return values (see Table S2).

## C. Honey collecting (Mbuti) [15]

**Return.** Ichikawa [15] documents Mbuti honey climbing during the honey season as achieving an average daily yield at 19kg across the 6 men in the honey camp: 14.25kg when accounting for waste in the form of wax (0.25% of weight). At 1272kJ per 100g [16], this represents 181,260kJ with negligible processing costs, and does not include that eaten on site during the expedition—often significant [17]. Failure rates are negligible: ‘once the Mbuti find out a beehive, they never fail to collect the honey, however difficult it may be’ [15 p.59], and are anyway accounted for in the daily averages used for yield calculations. The yield is achieved from a daily average of 3.75 honeybee hives and 0.30 stingless bee hives: 4.05 hives per day, with an average honey yield of 4.67kg (notably higher than the 3.15kg estimated by Bailey [17]).

**Cost.** Energy costs of locomotion include that of walking to and from the hives, as well as that of locating the hives in the first place, estimated at 1-2 hours in each case, so 2-4 hours per person per day for an average of 3 hours [15]. Using the energetic cost of 158J/min/kg quoted for Hadza men walking out of camp [11]—the average pace of which is documented to be 3.6-4.4 km/h, roughly comparable to that recorded for Mbuti men at 3.39km/h [17]—and assuming a bodyweight of 50kg, this adds to a total walking cost of 474kJ per hour per person: 8532kJ for the 18 total walking hours across the group assuming that all six individuals in the honey camp are working full hours every day, either together or in multiple groups. This does not, of course, account for the added energetic complexity of the Mbuti’s rainforest environment in comparison to the Hadza’s open savannah. The average weight of honey needing to be carried back to camp per person is 3.17kg (a sixth of the total 19kg), largely negligible with regards to estimated body weight, thus relocation costs are assumed to be included in the above total.

Regarding the climbing component, hives are found ‘several to thirty meters high from the ground’ [15 p.58]; an average height of 62.6ft [19.1m] according to Bailey’s [17] observations. Taking the 30m upper limit to account for any additional moving about in the tree this amounts to 243m of ascent and descent across the daily average of 4.05 hives. Using a climbing speed of 0.21m/s as for Twa foragers [18] this gives a COT of ~60J/kg/m based on values for rock-climbing [6]. Assuming equal velocities and COT for both ascent and descent, and bodyweight of 50kg, this amounts to 729kJ for the 243m of climbing required.

Finally, estimating an extraction cost of 2013kJ for hive cutting, based on the 497J/min/kg cost of chopping baobab among the Hadza [11]), a 50kg individual, and an estimate of 20 minutes chopping per hive out of the hour documented to be required for preparation, climbing, and chopping [15]. Total energetic costs for the party of six thus amount to 11,274kJ (1879kJ/person).

**Net.** Overall, this amounts to a net return of 169,986kJ (return:cost ratio of 16:1) over an average of 7 hours for a net return rate of 24,284kJ/hr (4047kJ/hr/person) for Mbuti honey collecting.

## D. Subaquatic spearfishing (Bajau) [19,20]

**Return.** Collective spearfishing by a team of four Bajau divers is documented to provide a catch of 10kg of fish per person in 4 hours [19]. Other data suggests a yield of 1-8kg/person for a typical dive session, with sessions lasting 2-9 hours, for a typical average of ~5 hours, including short breaks for water, food and relocations to new dive sites [21]. Yield can therefore be estimated at 1-10kg of fish over 4 hours of diving (5hr total expedition), including a variety of fish: ‘coral fish, blow fish, moray eels and octopuses’ [21]; according to Sather [22 p.95], ‘around forty varieties of fish, … include wrasse (Labridae and Scaridae), cardinalfish, damselfish, angelfish, and fusiliers. In deeper reefs, including areas of outer reef-face, catches also include butterfly perch, bass, parrotfish, bream, snapper, rock cod, surgeonfish, and mullet’. USDA data available for these species ranges from 161-273kcal/100g [16], for an average of ~217kcal, or 910kJ per 100g. Assuming a waste proportion of 30% [23] this amounts to 6370-63,700kJ for 1-10kg of fish.

**Cost.** Using energetic values documented for recreational breaststroke (22.2kJ/kg/hr; as a proxy for subaquatic diving) and treading water (14.6kJ/kg/hr) [24,25], and given a 60:40 time split respectively during Bajau spearfishing [20], we approximate the energetic cost of Bajau diving to be 19.2kJ/kg/hr: 3840kJ over four hours for a 50kg Bajau diver. An additional 1000kJ is added to account for the thermoregulatory and other physiological demands of prolonged diving at 26-29℃.

No additional extraction costs are relevant and given that this form of diving is documented to be conducted on shallow reefs we assume that it is carried out reasonably close to home (or traditionally from the houseboat), transportation costs by boat to the dive site are assumed to be negligible. Processing costs are also estimated to be negligible given the low energy demands of gutting and filleting fish, presumably conducted leisurely while seated back at home. Total energy costs estimated per individual are thus 4840kJ.

**Net.** Overall, this amounts to a net return of 1530-58,860kJ (return:cost ratio of 1-13:1) over 5 hours for a net return rate of 306-11,772kJ/hr (per person) for Bajau spearfishing.

**Note on turtle hunting.** A personal observation of the Bajau in Wakatobi National Park (March 2020) involved a Bajau fisherman opportunistically catching a large Green turtle by hand during a dive to ~10m deep, resulting in the termination a spearfishing session that had lasted approximately an hour. Representing ~50kg of edible weight [26] at 157kcal/100g [16] for an average Green turtle this approximates to 328.5MJ for only an hour of diving (costing 0.96MJ; see above): an extremely high net return rate of 218.4MJ/hr (assuming an additional half hour of travel/preparation; return:cost ratio of 342:1), before adjusting for processing costs and the caloric value of the additional four fish already caught. Such an event is, of course, atypical, if not ethnographically uncommon, with turtle encounters being irregular and unreliable, representing such a low success rate as to be opportunistic at best.

# Hunter-gatherer Load Carriage

***Table S3.*** *Selection of ethnographic examples of load carriage in hunter-gatherer terrestrial locomotor engagements. See Dataset S3, Supplementary Material for expanded list, references, full ethnographic passages and interpretative notes. Quote references refer to enumeration within dataset.*

| **Context/ Burden** | **Society** | **Weight (kg)** | **Distance/Time carried** | **Notes** | **Quote Reference** |
| --- | --- | --- | --- | --- | --- |
|  |  |  |  |  |  |
| General/ Unspecified | !Kung | 11-bodyweight (~40) | Bodyweight (~40kg) for 3 miles | Female, including child carriage | 6,16-17 |
|  | Aleut | 24-32 | 37-53km | Male & Female | 8 |
|  | Andamanese | 18 | 15 miles | Male | 9,10 |
|  | Aweikoma | 68 | - | Male | 11 |
|  | Lengua | 23-27 | 35 miles |  | 12 |
|  | Siriono | 27-32 (up to 91) | 91kg for 10 miles | Male & Female. 91kg anecdote male | 15 |
|  |  |  |  |  |  |
| Animal carcass | Aranda | Ext. 40-70kg | ‘for miles’ | Male. Large kangaroo | 1 |
|  | Mundurucu | 45 | 3 hrs | Male. Wild pig | 2 |
|  | Yurok | 68 | ‘half a day’ | Male. Quarter of an elk | 3 |
|  |  |  |  |  |  |
| Other foodstuffs | Callinago | 45 | ‘several miles’ | Male & Female. Bananas | 23 |
|  | Yokuts | 91 | - | Female. Acorns (and sometimes child) | 24 |
|  |  |  |  |  |  |
| Water | Aranda | 20 | 10-12 miles | Female | 29 |
|  |  |  |  |  |  |
| Sport | Timbira | 100 | 2-12km | 100kg log passed between runners as distance is run (typically 3km) | 30 |
|  |  |  |  |  |  |

# References

1. Rathkey JK, Wall-Scheffler CM. 2017 People choose to run at their optimal speed. *American Journal of Physical Anthropology* **163**, 85–93. (doi:10.1002/ajpa.23187)

2. di Prampero PE, Osgnach C. 1986 Energy Cost of Human Locomotion on Land and in Water. *International Journal of Sports Medicine* **7**, 55–72. (doi:10.1016/B978-0-12-814593-7.00009-8)

3. Capelli C, Pendergast DR, Termin B. 1998 Energetics of swimming at maximal speeds in humans. *European Journal of Applied Physiology and Occupational Physiology* **78**, 385–393. (doi:10.1007/s004210050435)

4. Vinetti G, Ferretti G, Francesco Lopomo N. In press. Energetics and mechanics of human breath-hold diving.

5. Halsey LG, Coward SRL, Crompton RH, Thorpe SKS. 2017 Practice makes perfect: Performance optimisation in ‘arboreal’ parkour athletes illuminates the evolutionary ecology of great ape anatomy. *Journal of Human Evolution* **103**, 45–52. (doi:10.1016/j.jhevol.2016.11.005)

6. Kozma EE, Pontzer H. 2021 Determinants of climbing energetic costs in humans. *Journal of Experimental Biology* **224**. (doi:10.1242/jeb.234567)

7. Halsey LG, Coward SRLL, Thorpe SKSS. 2016 Bridging the gap: parkour athletes provide new insights into locomotion energetics of arboreal apes. *Biology letters* **12**. (doi:10.1098/rsbl.2016.0608)

8. Morin E, Winterhalder B. 2024 Ethnography and ethnohistory support the efficiency of hunting through endurance running in humans. *Nat Hum Behav* , 1–11. (doi:10.1038/s41562-024-01876-x)

9. Vincent AS. 1985 Plant Foods in Savanna Environments: A Preliminary Report of Tubers Eaten by the Hadza of Northern Tanzania. *World Archaeology* **17**, 131–148.

10. Marlowe FW. 2010 *The Hadza: hunter-gatherers of Tanzania*. University of California Press. See https://ehrafworldcultures.yale.edu/cultures/fn11/documents/001/pages/3.

11. Kraft TS *et al.* 2021 The energetics of uniquely human subsistence strategies. *Science* **374**. (doi:10.1126/science.abf0130)

12. Glaub M, Hall CA. 2017 Evolutionary Implications of Persistence Hunting: An Examination of Energy Return on Investment for !Kung Hunting. *Human Ecology* **45**, 393–401. (doi:10.1007/s10745-017-9908-3)

13. Liebenberg L. 2006 Persistence hunting by modern hunter-gatherers. *Current Anthropology* **47**, 1017–1025. (doi:10.1086/508695)

14. Lee RB. 1979 *The !Kung San: Men, women, and work in a forager society*. New York: Cambridge University Press.

15. Ichikawa M. 1981 Ecological and sociological importance of honey to the Mbuti net hunters, Eastern Zaire. *African Study Monographs* **1**, 55–68. (doi:10.14989/67980)

16. USDA. 2024 USDA National Nutrient Database. See https://fdc.nal.usda.gov/fdc-app.html#/ (accessed on 11 May 2024).

17. Bailey R. 1991 *The Behavioral Ecology of Efe Pygmy Men in the Ituri Forest, Zaire*. Michigan: Ann Arbor.

18. Kraft TS, Venkataraman VV, Dominy NJ. 2014 A natural history of human tree climbing. *Journal of Human Evolution* **71**, 105–118. (doi:10.1016/j.jhevol.2014.02.002)

19. Schagatay E, Abrahamsson E. 2015 Three profitable non-extreme freediving strategies used by the Bajau-marine hunter-gatherers. In *1st International Conference on Sama Dilaut, Tawi-Tawi, Philippines*,

20. Schagatay E, Lodin-Sundström A, Abrahamsson E. 2011 Underwater working times in two groups of traditional apnea divers in Asia: the Ama and the Bajau. *Diving and Hyperbaric Medicine* **41**, 27–30.

21. Abrahamsson E, Schagatay E. 2014 A living based on breath-hold diving in the bajau laut. *Human Evolution* **29**, 171–183.

22. Sather C. 1997 *The Bajau Laut : adaptation, history, and fate in a maritime fishing society of south-eastern Sabah*. Oxford University Press.

23. Aswani S. 1998 The use of optimal foraging theory to assess the fishing strategies of Pacific Island artisanal fishers: A methodological review. *Traditional Marine Resource Management and Knowledge* **9**, 19–26.

24. Ainsworth BE, Haskell WL, Leon AS, Jacobs DR, Montoye HJ, Sallis JF, Paffenberger RS. 1993 Compendium of Physical Activities: classification of energy costs of human physical activities. *Medicine & Science in Sports & Exercise* **25**, 71–80. (doi:10.1249/00005768-199301000-00011)

25. Ainsworth BE *et al.* 2011 Compendium of Physical Activities: A Second Update of Codes and MET Values. *Med. Sci. Sports Exerc* **43**, 1575–1581. (doi:10.1249/MSS.0b013e31821ece12)

26. Bliege Bird RL, Bird DW. 1997 Delayed Reciprocity and Tolerated Theft: The Behavioral Ecology of Food-Sharing Strategies. *Current Anthropology* **38**, 49–78. (doi:10.1086/204581)
